# Dr. Clymer on the Mortality after Amputations

**Published:** 1842-09-03

**Authors:** Meredith Clymer


MEDICAL EXAMINER.

NEW SERIES.

No. 36.] PHILADELPHIA, SEPTEMBER 3, 1842. [Vol. I.

### ON THE MORTALITY AFTER AMPUTATIONS.

By Meredith Clymer, M. D.

In an analysis of Mr. Malgaigne's essay on the "Mortality after Amputations," recently published in this journal, it was proposed to compare his results with others obtained at different periods and in various places. A partial attempt to redeem this pledge will be made in the following pages. The practical utility of Mr. Malgaigne's paper was materially impaired by the omission of the cause of death in his cases. Unfortunately, the immediate lesion producing death has in no instance been stated in the imperfect data which form the ground-work of the present article. This all-important question may form a subject of inquiry at a future period.

After the battle of Fontenoy, Boucher declared that he lost nearly two-thirds of his amputations, and that the mortality was especially great in those of the leg. (Mem. de l'Acad. de Chirurgie, t. ii., p. 304.) Faure, in a paper read before the Academy at the same time, gave the preference to secondary amputations, and stated that in six cases of his own he was entirely successful, and that Read, of Valenciennes, out of 11 lost only 2. By immediate amputations, on the contrary, one-third was not saved; for after the battle of Fontenoy, out of 300 primary amputations, 30 or 40 only were successful. (Prix de l'Acad. de Chirurgie, t. iii., p. 492, 498, 518.) Bilguer, a surgeon of the Prussian army, affirms, that during the first part of the seven years war, that hardly more than one or two were saved out of all the amputations; and he asks whether the operation might not be in a measure abandoned. Benjamin Bell says, that even after the invention of the tourniquet more than one-half of the patients who submitted to amputation perished. He adds, however, that with the improvements then known, he does not believe that 1 in 20 perish in hospitals, and probably a smaller number in private practice.

In 1797, the late Baron Larrey stated that on the 27th and 30th brumaire he performed 13 primary amputations, and lost only 2. Mr. A. Blandin, his aide-major on that occasion, says, in a dissertation published at Strasburg, in 1803, that out of 5 [traumatic?] amputations you may save 3, under the most favourable circumstances.

In 1814, Mr. Roux reported a series of 22 great operations performed by him at la Charité. Of these 22, there were 2 of the arm, 1 of the forearm and 7 of the leg, and for these 10 he had 3 deaths. Of 12 amputations of the thigh there were 3 where reunion had not occurred, and of these 1 death; there remain 9 amputations of the thigh, with 4 deaths.

At the battle of New Orleans, Mr. Guthrie had 45 immediate amputations, 7 deaths; 7 secondary amputations, 5 deaths.

After the battle of Toulouse, the same surgeon had 47 immediate amputations, 9 deaths; 51 secondary amputations, 21 deaths. Thus, out of 150 amputations, 42 deaths, or more than one-fourth.

Mr. Marjolin, in 1814, at Salpetrière, had 14 amputations from gun-shot wounds, and saved but 1.

In September, 1829, Mr. Emery read before the Academy of Medicine a note from Mr. Del Signore, surgeon of the Egyptian navy at the battle of Navarino;—the results were as follows:

| | |
|---|---|
| 31 immediate amputations, | 1 death. |
| 29 secondary amputations, from 2d to 3d day, | 11 deaths. |
| 8 secondary amputations, from 10th to 12th day, | 2 deaths. |

In 1830, Dupuytren gave in a clinical lecture the following results of his practice:

| | |
|---|---|
| 30 amputations with immediate reunion, | 6 deaths. |
| 29 amputations with immediate reunion, | 9 deaths. |

Larrey, in one of his publications says, after 20 years of war, that he saved three-fourths of his amputations.

Mr. Paul Dubois (Lancette Française, 9 Mars, 1840,) states that his father performed, in the course of three years, at the Maison Royale de Santè at Paris, 28 amputations; 13 of the thigh, 12 of the leg, 2 of the arm, and 1 of the forearm. In 3 cases immediate reunion did not take place, no deaths. In 25 cases, with union by the first intention, 3 deaths. Mortality one-tenth.

From Mr. Malgaigne's paper we learn that the mortality in the Polish army, during the insurrection of 1831, was frightful, nearly all the amputations dying. He does not state the cause of death, but leads us to infer that it was from the shock sustained by the system, and metastatic abscess. He says death rarely took place from hæmorrhage. The surgeons of the Polish army at that time were from every country of Europe, and, of course, the practice was very varied.

Mr. Menière, in his work on the operations at the Hôtel Dieu, during the revolution of July, 1830, gives the following as the results of the great traumatic amputations:

| | | | |
|---|---|---|---|
| 11 | amputations of the thigh, | | 9 deaths. |
| 3 | " | " leg, | 3 " |
| 10 | " | " shoulder and arm, | 5 " |

Thus, in Dupuytren's hands, out of 10 amputations of the arm, one-half died, and out of 14 of the inferior extremity, 12 died: out of 24 amputations, 17 died, whilst Larrey states his mortality at the same period to have been 7 out of 16.

Roux, at la Charité, for 10 immediate amputations had 3 deaths; 3 amputations of the inferior extremity, 1 death; for 7 of the superior extremity, 2 deaths; and 4 deaths for 4 secondary amputations. Total, 14 amputations, 7 deaths.

After the *émeute* of 1832, Richerand, at St. Louis, in 10 amputations of the inferior extremity had 8 deaths; 2 remaining under treatment; 5 of the superior extremity, 3 deaths, and 2 under treatment. Total, (at least,) 11 deaths out of 15 cases. They were nearly all primary amputations, and all the result of gun-shot wounds.

In resuming we find 69 amputations, 32 deaths. For the inferior extremity alone, 40 amputations, and 28 deaths.

Mr. Baudens, in the expedition against Constantine, out of 29 amputations lost 24.

According to Mr. Malgaigne, the mortality at the Enfans Malades at Paris, among the children after amputation, is fearfully great.

So far our inquiries have been directed solely towards the mortality after amputation following gun-shot wounds of the extremities. We shall now refer to the results which have been obtained in the various civil hospitals of Europe and this country.

During the year ending August, 1840, Mr. Velpeau, out of 18 great amputations, lost but 1. He stated in his lecture at that time, that in a previous year, out of 26 amputations, he lost only 2. In another year he lost 6 out of 21, and in another 4 out of 19. Dr. W. Poyntell Johnston states (Med. Examiner, O. S., vol. iii, p. 300,) that out of 11 capital amputations performed by Velpeau during the clinical year, ending August, 1837, 1 only died—an amputation through the knee-joint.

Chelius, the Professor of Surgery at Heidelberg, saved 27 out of 29 amputations.

In 1840, at the Hôtel Dieu, in the service of Mr. Roux, there were 18 capital amputations and 8 deaths, distributed as follows:—of the thigh, 8 cases and 4 deaths; of the leg, (at the place of election,) 2 cases and 1 death; of the leg, (above the malleoli,) 4 cases and 1 death; of the arm, 3 cases and 2 deaths; of the forearm, 1 case and 0 death.

In 1841, in the same service, there were 17 great amputations and 8 deaths, distributed as follows:—of the thigh, 8 cases and 5 deaths; of the leg, (at the place of election,) 5 cases and 2 deaths; of the leg, (above the malleoli,) 1 case and 1 death; of the forearm 3 cases and 0 death.

These two years give 35 great amputations, and result in 16 deaths, and 18 recoveries. This result may be considered as favourable, in comparison with the preceding years; for since 1836, there have been at the Hôtel Dieu, in all the surgical services united, 178 amputations, with 104 deaths and 74

recoveries. Of these 178 amputations, there were 63 amputations of the thigh, of which there were 43 deaths, and 20 recoveries.*

The above numbers are too few to establish any rigorous results, but the progression would be from the most dangerous to the least dangerous amputation—arm, thigh, leg, and forearm. The importance of distinguishing the lesion necessitating the amputation is acknowledged; in the above series we find the traumatic amputations were 11, and 9 deaths; they are distributed as follows: thigh 4, 4 deaths; leg 3, 3 deaths; arm 2, 2 deaths; forearm 2, 0 deaths. Of the pathological amputations there were 21, and 7 deaths; distributed thus: thigh 12, deaths 5; leg 9, 2 deaths; arm 1, 0 deaths; forearm 2, 0 deaths.

From the statistics of the Glasgow Infirmary, published by Dr. Lawrie, we find that out of 276 great amputations, there recovered 176, or 63.7 per cent., and died, 100, or 36.3 per cent.; the deaths to the recoveries being as 1 to 2.75. Of these there were 153 pathological amputations, of which number 118, were cured, or 77.1 per cent., and 35 died, or 22.9 per cent. Of the traumatic amputations, 77 were primary, of which 39 died; and 46 secondary, of which 26 died. The proportionate mortality among the males to that among the females, was as 1.6 to 3.3.

According to Dr. Norris, the general mortality in the Pennsylvania Hospital, from amputations, was 1 in 3 7-11ths. In traumatic amputations the mortality was 1 in 3 2-11ths, in chronic diseases it was 1 in 6¼. Immediate traumatic amputations were less fatal than secondary ones, the mortality in the former being 1 in 3 2-11ths, whilst in the latter it was 1 in 2 6-7ths. Amputation of the lower member was much more fatal than that of the superior, the mortality after the former, being 1 in 2 15-16ths; after the latter, 1 in 6 2-5ths. Dr. N. says that during a period of 10 years, none of the lesser amputations were attended by death.

* Experience seems daily to prove the comparative safety of resection over amputation, in such cases as admit of the former operation. Its applicability is confessedly limited; and however brilliant and useful have been its results, when applied to the superior extremity, its danger to the inferior member must be generally admitted. Within the last two years Mr. Roux has performed at the Hôtel Dieu resection of the elbow-joint 6 times, and has had but 1 death; this latter occurring on the sixteenth day, from an extensive erysipelas. Since August, 1840, Mr. Roux has replaced the incision in H, employed by surgeons generally since Moreau in this operation, by one in T, made on one side of the joint. This modification, Mr. R. thinks, facilitates the dressing, without causing the slightest movement in the articulation.

A year since, at the Hôtel Dieu, a girl of 19 years of age was to be seen, who had submitted to resection of the elbow-joint fourteen years previously, and who was then able to use perfectly her arm and hand. MM. Maunoury and Thore mention recently, in the Gazette Medicale, a similar case, seen two years subsequent to the operation, where motion was entirely reëstablished, and the cicatrix solid.

Of 124 amputations, reported by Dr. Hayward, of the Massachusetts General Hospital, there were 37 deaths, or a fraction more than 1 in 4. Dr. H.'s table would show the greater chance of success in amputation for chronic disease, than after injuries. In amputation of the thigh Dr. H. states that more than one-fourth died; whilst in amputation of the leg, the mortality was little more than 1 in 5.

According to the results of the statistics of Mr. B. Phillips, (Med. Examiner O. S. vol. i., page 290,) there was an average mortality of 23½ per cent.

The rate of mortality for the ages, between 9 and 20, according to Dr. Lawrie, is by far the most favourable; whilst that from 60 to 50 is the least so. These results correspond with those of Drs. Norris and Hayward; under 20, both losing only 1 in 13. Mr. Malgaigne's data as already has been said (p. 487) are insufficient to warrant any conclusions on this point.

Of 551 cases of secondary amputations, performed by Mr. Guthrie, 265, or nearly one-half, died; of 291 primary amputations, 24 died, or nearly 1 in 12. All were in consequence of gunshot wounds.

Army surgeons seem to have established as a maxim, the necessity of amputation in all cases of gunshot wounds in the lower extremity. Mr. Ribes expresses, in his essay, his solemn conviction that any delay necessarily endangers the life of the wounded person, and as far as the middle portion of the thigh is concerned, the late Baron Larrey coincides in this opinion. Mr. Malgaigne's sad experience in Poland, induces him to dissent to this doctrine, and he mentions the following incident, as bearing on the question. At a surgical concours, where this point was discussed, Mr. Malgaigne being the interrogator, he expressed the opinion that if his own thigh were fractured by a shot, he would not, weighing all chances, submit to an amputation. Mr. Marjolin, the President, on hearing this avowal, exclaimed with some vehemence, "*nor I either.*"

The opinion of Mr. Ribes would appear to have been formed from the following circumstances. On his return to Paris, in 1814, out of 4,000 patients at the Invalides, he could not find a single case of gunshot wound of the femur, but he saw one case where the thigh had been amputated for this injury. But as it was at that time the custom in the French Army to amputate in all such cases, might it not be legitimately concluded that all had died who had submitted to amputation? From 1814 to 1822, he mentions having seen seven, who had survived the injury, but says nothing of having met any who had survived the operation. Mr. Malgaigne, in his paper, so frequently alluded to, states that he saw, at Warsaw, two cases where the femur was solidly, though viciously consolidated, after this injury, and adds, that he could cite others, which most unaccountably he does not do.

At the seige of Genoa, the leg of Marshal Soult was fractured by a shot; he resisted all the instances made by his surgeon for immediate amputation. He has since made 20 campaigns, and mounts a horse with ease, by means of the condemned member, and is able to remain, at the age of 73, from six to eight hours in the saddle.

*August* 28, 1842.

---

